# Screening New Blood Indicators for Non-alcoholic Fatty Liver Disease (NAFLD) Diagnosis of Chinese Based on Machine Learning

**DOI:** 10.3389/fmed.2022.771219

**Published:** 2022-06-09

**Authors:** Cheng Wang, Junbin Yan, Shuo Zhang, Yiwen Xie, Yunmeng Nie, Zhiyun Chen, Sumei Xu

**Affiliations:** ^1^Applied Math Department, China Jiliang University, Hangzhou, China; ^2^The First Affiliated Hospital of Zhejiang Chinese Medical University, Hangzhou, China; ^3^Key Laboratory of Integrative Chinese and Western Medicine for the Diagnosis and Treatment of Circulatory Diseases of Zhejiang Province, Hangzhou, China; ^4^Gastroenterology Department, Zhejiang Provincial Hospital of Chinese Medicine, Hangzhou, China; ^5^Department of General Practice, Zhejiang Provincial Hospital of Chinese Medicine, Hangzhou, China

**Keywords:** non-alcoholic fatty liver disease (NAFLD), TPA, PAI-1, machine learning, support vector machine (SVM), predictive model

## Abstract

**Background:**

The prevalence of NAFLD is increasing annually. The early diagnosis and control are crucial for the disease. Currently, metabolic indicators are always used clinically as an auxiliary diagnosis of NAFLD. However, the prevalence of NAFLD is not only increased in obese/metabolic-disordered populations. NAFLD patients with thin body are also increasing. Only using metabolic indicators to assist in the diagnosis of NAFLD may have some deficiencies. Continue to develop more clinical auxiliary diagnostic indicators is pressing.

**Methods:**

Machine learning methods are applied to capture risk factors for NAFLD in 365 adults from Zhejiang Province. Predictive models are constructed for NAFLD using fibrinolytic indicators and metabolic indicators as predictors respectively. Then the predictive effects are compared; ELISA kits were used to detect the blood indicators of non-NAFLD and NAFLD patients and compare the differences.

**Results:**

The prediction accuracy for NAFLD based on fibrinolytic indicators [Tissue Plasminogen Activator (TPA), Plasminogen Activator Inhibitor-1 (PAI-1)] is higher than that based on metabolic indicators. TPA and PAI-1 are more suitable than metabolic indicators to be selected to predict NAFLD.

**Conclusions:**

The fibrinolytic indicators have a stronger association with NAFLD than metabolic indicators. We should attach more importance to TPA and PAI-1, in addition to TC, HDL-C, LDL-C, and ALT/AST, when conducting blood tests to assess NAFLD.

## Introduction

Non-alcoholic fatty liver disease (NAFLD) has become one of the most common liver diseases, affecting about 25% of the general population worldwide, in which Asia (27%) has higher prevalence rates comparing with North America (24%) and Europe (24%) (1, 2). As the largest country in Asia, the prevalence of NAFLD in China is also increasing annually (3). NAFLD is closely related to metabolism (4), so metabolic indicators are often used to assist the diagnosis of NAFLD in the clinic. Total cholesterol (TC), high-density lipoprotein cholesterol (HDL-C), low-density lipoprotein cholesterol (LDL-C), alanine transaminase/aspartate transaminase (ALT/AST), and body mass index (BMI), and other indicators of metabolism, are all regarded as important factors related to the risk of NAFLD and contribute to the diagnosis (5–7). However, there are still some shortcomings in using only metabolic indicators as predictors of NAFLD. A paper published in “The Lancet” (8) pointed out that even thin people are not immune to fatty liver disease. Of the total incidence of NAFLD, 40% of patients with NAFLD had normal BMIs (18.5–23.9), and 20% of non-obese people had NAFLD. This inspired us to find more evidence in addition to metabolism for the accurate diagnosis of NAFLD. Our research focused on the fibrinolytic indicators.

The physiological balance of TPA/PAI-1 plays an essential role in regulating blood patency and preventing atherosclerosis (9). Also, plasma TPA and PAI-1 are associated with many metabolic diseases including NAFLD, heart disease, and diabetes mellitus (DM) (10–12). Jin found that plasma PAI-1 levels were significantly increased in children with increased severity of steatosis, lobular inflammation, ballooning, and fibrosis (13). Furthermore, PAI-1 was strongly correlated with plasma lipids and insulin resistance indices (13). By analyzing 210 Taiwanese NAFLD patients and 420 gender- and age-matched control groups, Chang found that based on univariate analysis, TG, BMI, LDL, HDL, ALT, AST, TPA, and PAI-1 are all related to NAFLD (14). However, less research provided good predictive accuracy for NAFLD diagnosis based on fibrinolytic or metabolic indicators. And there was little research on comparing the impacts of these indicators on NAFLD diagnosis quantitatively.

In this study, we applied machine learning (ML) which has been increasingly used in the field of liver disease and liver transplantation (15) to construct the predictive models for NAFLD based on those blood indicators, and obtained good predictive accuracy. It also compares the accuracy of prediction, looking for which indicators are more suitable for NAFLD diagnosis, fibrinolytic indicators, or metabolic indicators? We collected the datasets of 365 patients who had blood tests and NAFLD labels from the Traditional Chinese Medicine hospital of Zhejiang Province. The support vector machine (SVM) method was applied to the dataset to construct a predictive model for NAFLD based on the indicators above. SVM has been used to identify molecular markers of hepatocellular carcinoma (HCC) (16), but no one has yet used it to screen NAFLD auxiliary diagnostic indicators. We compared the prediction accuracy for NAFLD diagnosis based on fibrinolytic indicators (TPA and PAI-1) with the prediction accuracy based on metabolic indicators (TC, HDL-C, LDL-C, ALT/AST), screened the more accurate one.

## Materials and Methods

### Screen and Compare Diagnostic Indicators

#### Subjects

##### Ethics Statement

Ethics statement Written informed consent was obtained from each participant, and the study was approved by the Committee for the protection of human subjects of The First Affiliated Hospital, Zhejiang Chinese Medical University. The corresponding ethical approval code (2018-K-061-01).

##### Inclusion Criteria

This study investigated 365 adult individuals aged 18–65 on whom we had complete data. They are from the health examination center of the Traditional Chinese Medicine hospital of Zhejiang Province. The following subjects were excluded:

(1) pregnant or lactating women;
(2) who has one of the following diseases: heart, brain, blood, lung, kidney, endocrine, mental, viral hepatitis, tuberculosis, AIDS, scarlet fever, drug-induced hepatitis, autoimmune liver disease, Wilson's disease, and liver cancer;
(3) who has taken anticoagulants in the last half month.

365 adult individuals who met the inclusion and exclusion criteria were divided into the Normal group (*n* = 99) and the NAFLD group (*n* = 299) according to the B-ultrasound results for follow-up analysis. Detailed clinical data can be found in Supplementary Table 1.

#### Methods

The following variables are included in our model: gender, age, body mass index (BMI), body height, TPA, PAI-1, TC, HDL-C, LDL-C, ALT/AST. These input variables were linearly scaled to the range [0, 1] and were mapped into a high-dimensional feature space. For details, see Table 1.

**Table 1:** The characteristic clinical data between the NAFLD and non-NAFLD patients.

**Characteristic**	**Non-NAFLD (*n =* 99)**	**NAFLD (*n =* 266)**
Gender (*n*, %)	Female (79, 79.8%)	Female (55, 20.7%)
	Male (20, 20.2%)	Male (211, 79.3%)
Age, meidan (IQR)	40 (35, 48)	42 (37, 51)
tpa, meidan (IQR)	5,956.28 (3,923.5, 8,163.93)	9,239.07 (6,383.61, 11,975.68)
pai-1, meidan (IQR)	15,384.16 (13,605.38, 18,530.64)	32,095.67 (23,665.17, 37,275.04)
PAI-1/TPA, meidan (IQR)	2.66 (1.88, 3.88)	3.31 (2.22, 5.25)
BMI, meidan (IQR)	22.08 (20.07, 23.86)	26.37 (24.69, 28.31)
TC, meidan (IQR)	4.19 (3.8, 4.77)	4.8 (4.27, 5.44)
TG, meidan (IQR)	0.9 (0.68, 1.12)	1.68 (1.19, 2.37)
HDL-C, meidan (IQR)	1.49 (1.29, 1.67)	1.09 (0.97, 1.28)
LDL-C, meidan (IQR)	2.08 (1.77, 2.44)	2.65 (2.18, 3.1)
ALT, meidan (IQR)	14 (11, 18)	26 (19, 38)
AST, meidan (IQR)	16 (14, 18)	21 (17, 26)
AST/ALT, meidan (IQR)	1.17 (0.94, 1.34)	0.8 (0.63, 1)

*NAFLD, metabolic associated fatty liver disease; IQR, interquartile range*.

Comparisons between the two groups (NAFLD vs. non-NAFLD) were conducted using Student *t*-tests for continuous variables and Pearson tests for categorical variables.

SVM methods were taken to construct predictive models for NAFLD. SVM is a very popular supervised machine learning classifier widely used in classification or discrimination analysis. For non-linear and complicated relationships in high-dimensional variables, SVM is usually more effective than Logistic and other ordinary statistical methods. In this research, the relationship between NAFLD and blood indicators is complicated and no regular mathematical function can precisely describe the mechanisms between NAFLD and blood indicators. So SVM is suitable for our topic.

We introduce briefly the idea of svm. Let *X*_*i*_ denote the input variables such as TPA, PAI-1, BMI and so on in our case, and *y*_*i*_ denote the lable of each sample. The purpose of SVM model is to find a function $\omega^{T}X_{i}+b$ to predict the lable as accurate as possible. It implement the following optimal problem to solve the function.


$$\begin{array}{l} \;\underset{\omega ,b}{\min}\frac{1}{2}∥\omega {∥}^{2}+C\sum_{i=1}^{L}\xi_{i},\\ s.t.\;y_{i}\left(\omega^{T}X_{i}+b\right)\geq 1-\xi_{i},\;\xi_{i}\geq 0; \end{array}$$

We attached different weights to the two categories, i.e., the objective function was replaced by


$$\underset{\omega ,b}{\min}\frac{1}{2}∥\omega {∥}^{2}+C\sum_{i=1}^{L}w_{i}\xi_{i}.$$

Each ω_*i*_ for normal cases (NAFLD label *y*_*i*_ = 1) had a common value denoted by $\bar{\omega }$, while each ω_*i*_ for NAFLD cases (NAFLD label *y*_*i*_ = −1) had another common value denoted by ω. We adjusted the value of $\bar{\omega }$ and ω based on particular cases. For practical problems, we take $\bar{\omega }$ > ω if we believe the risk induced by misclassifying a label -1 sample as label 1 is larger than that induced by misclassifying a label 1 sample as label 0. Otherwise, we take $\bar{\omega }$ < ω.

**Figure 1:**
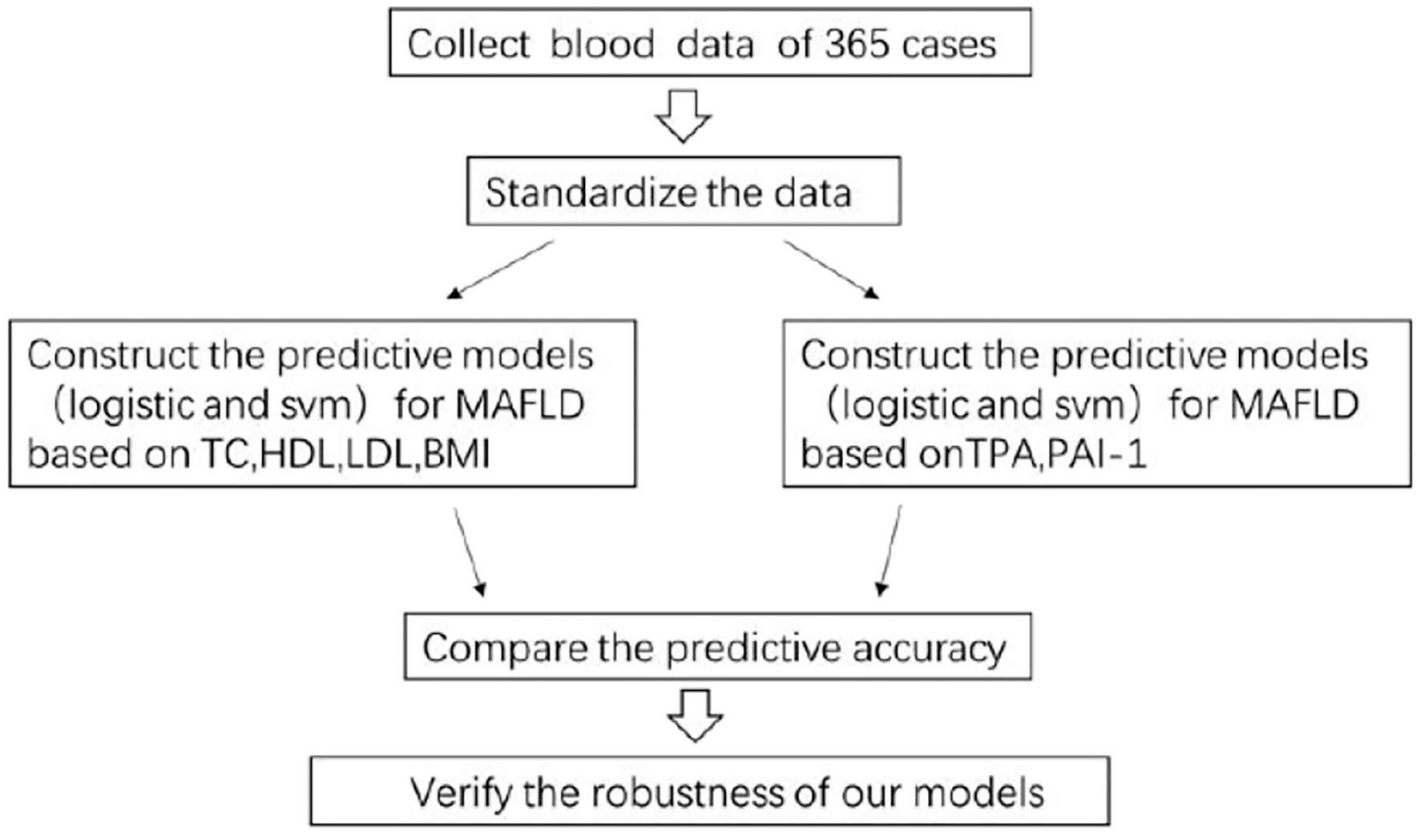
The technical line of our research.

We used the LIBSVM package (http://www.csie.ntu.edu.tw/~cjlin/libsvm) to implement the soft margin SVM model. The Gauss kernel function was applied in our study, which gives the highest accuracy for our test. The receiver operating characteristic (ROC) curve was used to assess the predictive performance of our SVM models. We generated the ROC curve by drawing the true-positive rates vs. false-positive rates over a range of thresholds. Each threshold is a cutoff, if an individual's output probability in the SVM is greater than this cutoff, he is judged as NAFLD, otherwise, he is judged as non-NAFLD. For each threshold, we calculated a pair of true-positive rates and false-positive rates. When the thresholds ranged stepwise from 0 to 1 by step size 0.01, we obtained the whole ROC curve. The area under the curve (AUC) was used as a measure of the predictive performance of our SVM models. The following Figure 1 is the technical line of machine learning.

## Results

### The Results of Screen and Compare Diagnostic Indicators

#### Basic Statistical Analysis Results of TPA and PAI-1

In the dataset of 365 cases, the patients' ages ranged from 25 to 65 years old, 266 patients had NAFLD, and 99 patients were normal. We used a *t*-test to compare the TPA, PAI-1, and TPA/PAI-1 between the NAFLD group and the non-NAFLD group. TPA, PAI-1, and TPA/PAI-1 exhibited a significant difference (*P* < 0.05) between the two groups. The mean of TPA and PAI-1 in the NAFLD group was higher than that in the non-NAFLD group. However, the mean of the ratio TPA/PAI-1 in the non-NAFLD group was significantly higher than that in the NAFLD group (*P* < 0.05). The results are summarized in Table 2. The results showed that no matter whether it was the plasm level of TPA, PAI-1, or TPA/PAI-1, there were significant differences between the NAFLD and the non-NAFLD patients, which suggests that the plasma levels of TPA, PAI-1, or TPA/PAI-1 have the potential to be regarded as indicators for NAFLD diagnosis.

**Table 2:** Comparison of TPA1, PAI-1 between the NAFLD and non-NAFLD patients.

	**Group**	**Mean ±std**	***t*-statistic**	***P***
TPA1	NAFLD group	9,596.64 ± 4,190.25	7.76	0.000
	Non-NAFLD group	6,311.64 ± 3,344.76		
PAI-1	Group	Mean ± std	*t*-statistic	*p*
	NAFLD group	31,438.98 ± 8,124.59	22.16	0.000
	Non-NAFLD group	16,589.63 ± 4,461.35		
TPA/PAI-1	Group	Mean ± std	*t*-statistic	*p*
	NAFLD group	0.33 ± 0.18	−2.59	0.01
	Non-NAFLD group	0.40 ± 0.22		

*TPA, plasma plasminogen activator; PAI-1, plasminogen activator inhibitor-1; Std, standard deviation*.

#### Predictive Results for NAFLD Using Metabolic Indicators as Predictors

First, we standardized the TC, HDL-C, LDL-C, and BMI data. In order to better assess the performance of the SVM predictive model for NAFLD, we first constructed the Logistic aggression model to predict NAFLD using the standard TC, HDL-C, LDL-C, and BMI data. The Logistic model was implemented in SPSS 25.0 but the predictive accuracy was <30%. Then we used the standard data to construct SVM predictive models for NAFLD. The results of the SVM model were summarized in Table 3. Error_1 was used to denote the misclassification rate of predicting normal samples as NAFLD samples and Error_2 was used to denote the misclassification rate of predicting NAFLD samples as normal samples. The results show that in the experiment, the accuracy of the SVM model is much higher than that of the Logistic model, suggesting the SVM model is more suitable for the predictive study.

**Table 3:** Prediction performance using BMI, TC, HDL-C, and LDL-C as factors.

	**Error_1**	**Error_2**	**Total accuracy**
Training set	37%	5%	85.35%
Testing set	39%	8%	85.87%

*BMI, body mass index; TC, total cholesterol; HDL-C, high-density lipoprotein cholesterol; LDL-C, low-density lipoprotein cholesterol*.

#### Predictive Results for NAFLD Using Fibrinolytic Indicators as Predictors

As above, we first constructed the Logistic model using the standardized TPA and PAI-1 as predictors but found that the predictive accuracy was not more than 40%. Next, we constructed an SVM model using the standardized TPA and PAI-1 as input variables. And we found that the predictive accuracy was much higher than that of the Logistic model. The results are shown in Table 4. These results suggest that, similar to metabolic indicators, the use of the SVM model to predict fibrinolytic indicators is more accurate.

**Table 4:** Prediction performance using TPA and PAI-1 as factors.

	**Error_1**	**Error_2**	**Total accuracy**
Training set	15%	4%	92.58%
Testing set	10%	8%	91.48%

*TPA, plasma plasminogen activator.*

*PAI-1, plasminogen activator inhibitor-1*.

#### The Comparison of the Prediction of Metabolic and Fibrinolytic Indicators

**Figure 2:**
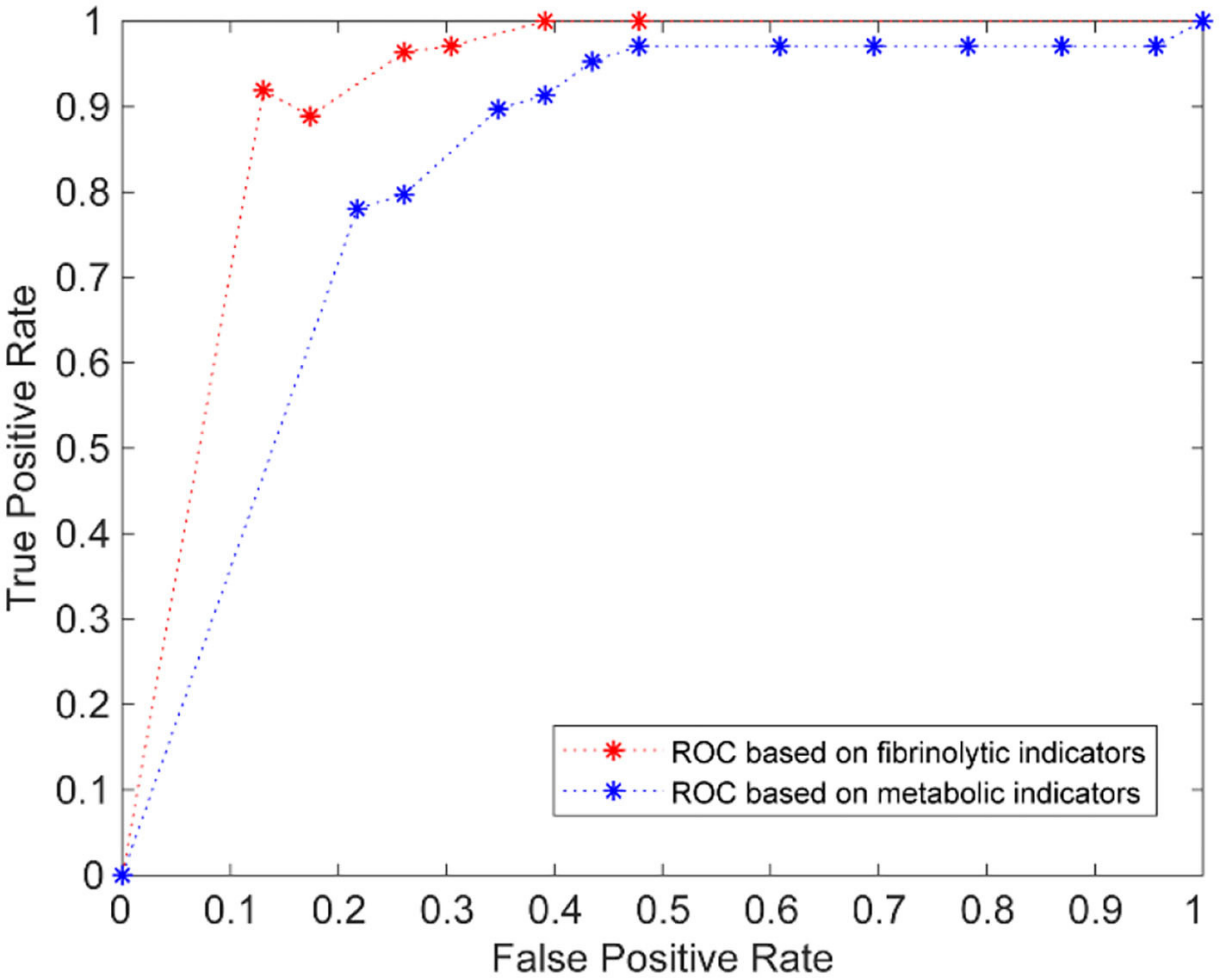
The comparison of ROC curves of SVM based on fibrinolytic indicators and metabolic indicators.

Interestingly, we found that the predictive accuracy based on TPA and PAI-1 was significantly higher than that based on TC, HDL-C, LDL-C, and BMI. To better see the difference, we drew the two ROC curves (Figure 2). The red curve is the ROC curve using TPA and PAI as predictors and the AUC is 0.91; the blue curve is the ROC curve using TC, HDL-C, LDL-C, and BMI as predictors and the AUC is 0.75. The difference was obvious. From the above results, we inferred that TPA and PAI-1 are more suitable than TC, HDL-C, and LDL-C for predicting NAFLD. TPA and PAI-1 have deeper links with NAFLD than TC, HDL-C, and LDL-C do.

We also want to know whether TC, HDL-C, and LDL-C can be complementary to TPA and PAI-1 to achieve better prediction results (in other words, whether TPA and PAI-1 miss some valuable information contained in the TC, HDL-C, and LDL-C data) when predicting NAFLD. Thus, we combined the TPA and PAI-1 data with the TC, HDL-C, and LDL-C data to construct an SVM model. The predictive results are in Table 5. The results show that compared with the prediction performance using BMI, TC, HDL-C, and LDL-C as factors, after adding TPA and PAI-1, the prediction accuracy of metabolic indicators is greatly improved. However, the prediction accuracy of the SVM model did not increase significantly compared with TPA and PAI-1 alone as a predictor. These indicate that the blood levels of TPA and PAI-1 can be regarded as highly effective indicators to assist the diagnosis of NAFLD, independent of metabolic indicators.

**Table 5:** Prediction performance using TPA, PAI-1, TC, HDL-C, LDL-C, and BMI as indicators.

	**Error_1**	**Error_2**	**Total accuracy**
Training set	13%	4%	93.57%
Testing set	8%	5%	92.65%

#### The Robustness of Our SVM Model

**Figure 3:**
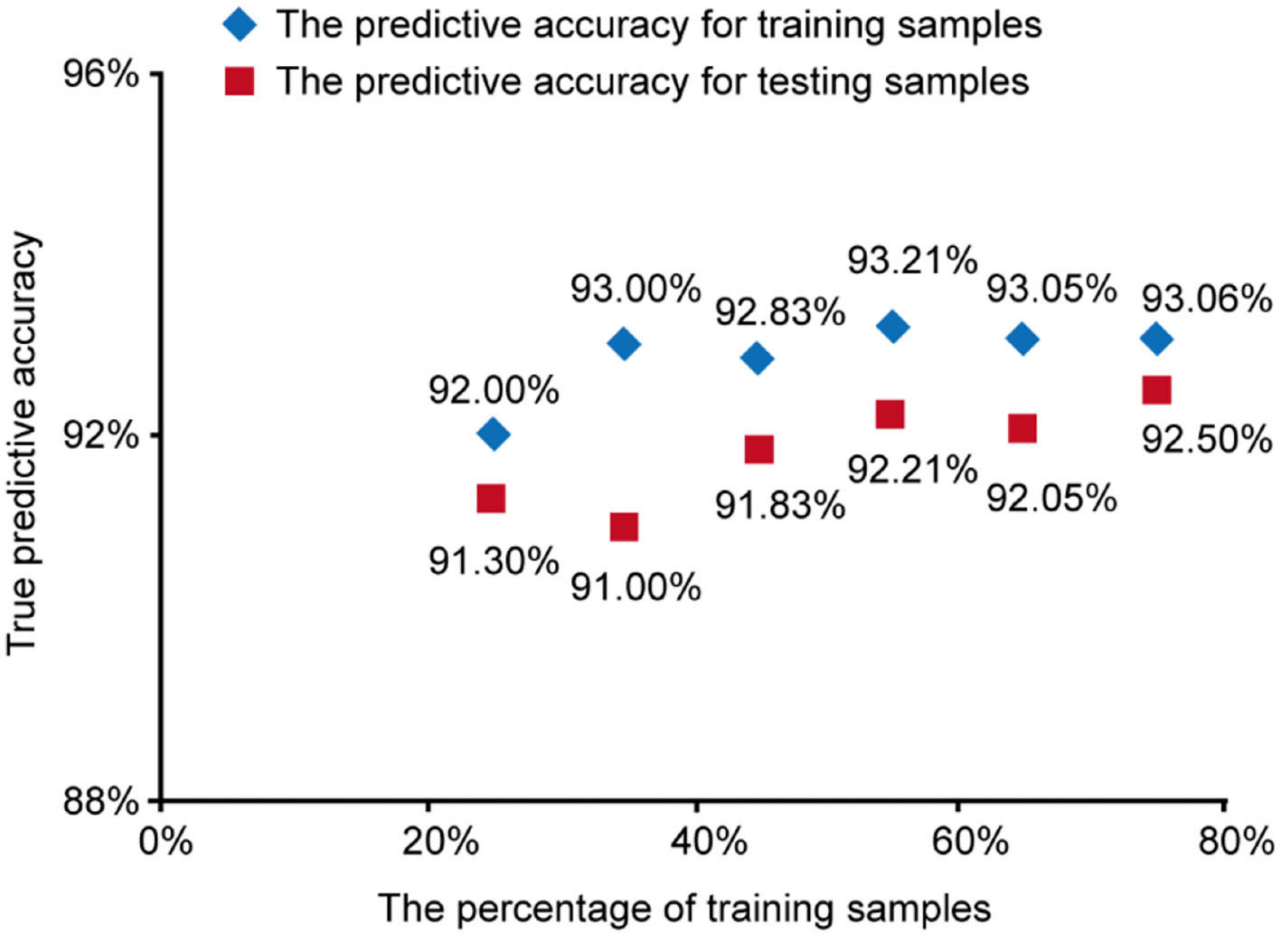
The predictive accuracy for training samples and testing samples vs. the percentage of training samples.

To check the robustness of our SVM model based on TPA and PAI-1, we took different percentages of training samples in the total 365 cases. The percentage varied from 25 to 75% and we obtained the corresponding accuracy of predicting NAFLD as in Figure 3.

The results show that in different percentages of training samples, the prediction accuracy of training samples and test samples are both high (over 90%), which indicates our SVM model based on TPA and PAI-1 was stable and trustable.

## Discussion

NAFLD is characterized by the significant accumulation of lipids, such as TG, TC, HDL- C, LDL-C in hepatocytes and serum, indicating that altered lipid metabolism is crucial in the pathogenesis of NAFLD (17). NAFLD is a broad-spectrum disease, including simple steatosis in the early stage, non-alcoholic steatohepatitis, liver fibrosis, cirrhosis, and even liver cancer in the late stages (18). The pathogenesis of NAFLD has been widely accepted by the “multiple-hit” hypothesis because NAFLD pathogenesis involves many influence factors, such as diet, genetic, environmental, and metabolism that progress through different stages during the occurrence and development of NAFLD (19). Although the number of patients with NAFLD is large and the harm is great, the exact mechanism of NAFLD is still unclear.

TPA and PAI-1 are mainly a pair of biological regulatory factors synthesized and secreted by vascular endothelial cells. Fibrinolytic system balance is affected by many factors, such as blood lipids, blood glucose, stress, gender, and age. And it is associated with obesity, insulin resistance, diabetes, dyslipidemia, and premature aging (13, 20, 21), which all are coexisting conditions of NAFLD. All this suggests that TPA and PAI-1 may be related to the metabolism and hepatic functions of NAFLD patients, but the specific mechanism is currently unknown.

What's more, by reviewing the literature, we found that the imbalance of TPA and PAI-1 activity is of great significance in metabolism, chronic liver disease and has different manifestations in different stages of the disease (22). And Based on our results, the prediction accuracy of NAFLD using TPA and PAI-1 as predictors was higher than that using TC, HDL-C, LDL-C, and ALT/AST as predictors. These discoveries all further suggest that the plasma level of TPA and PAI-1 may be used as new indicators for the diagnosis of NAFLD.

Nowadays, the gold standard for the NAFLD diagnosis is the liver biopsy (23), But liver biopsy cannot be used routinely, since it is an invasive and expensive procedure. In clinical diagnosis, we often use liver B ultrasound combined with clinical symptoms and metabolic indicators to diagnose NAFLD (24). Through the study, we propose that changes to the fatty liver fibrinolytic system are one of the key links in NAFLD progress. The change to the fibrinolytic system was even more significant for NAFLD than the internal metabolic indices such as liver and kidney function. Therefore, we propose that TPA and PAI-1 should be included in normal physical examinations. Further, studies of fibrinolytic activity and drug development may be important for understanding the mechanism and treatment of NAFLD. Based on the perspective of the fibrinolytic system, in-depth discussion on its prediction of NAFLD may play an important role in improving the mechanism of NAFLD.

However, this study also has some shortcomings. In this observation object, our inclusion criteria are B ultrasound diagnosis, so it is difficult to distinguish the stratification of NAFLD disease and Unable to analyze changes in the fibrinolytic system during the disease progression. Therefore, in the following study, we look forward to using H1-MRS, controlled attenuation parameter, through human or animal and cell experiments to analysis of its internal mechanism.

## Conclusion

In summary, TPA and PAI-1 are also effective indicators for the Chinese to assist in the diagnosis of NAFLD. Its diagnostic accuracy may be higher than metabolic related indicators. We do hope that this study can promote the further development of clinical NAFLD diagnosis and provide valuable guidance for the non-invasive diagnosis of NAFLD.

## Data Availability Statement

The original contributions presented in the study are included in the article/Supplementary Materials, further inquiries can be directed to the corresponding author/s.

## Ethics Statement

The studies involving human participants were reviewed and approved by the committee for the protection of human subjects of the First Affiliated Hospital, Zhejiang Chinese Medical University. The patients/participants provided their written informed consent to participate in this study. Written informed consent was obtained from the individual(s) for the publication of any potentially identifiable images or data included in this article.

## Author Contributions

CW, JY, and YN participated in drafting the manuscript. SZ, YX, and YN provided technical assistance. CW and JY revised the manuscript. All of the authors read and approved the final manuscript.

## Funding

This work was supported by Natural Science Fundation of Zhe Jiang Province (No. LY21H270009); Chinese Medicine Science and Technology Plan of Zhejiang Province (2021ZA062 and 2020ZB065).

## Conflict of Interest

The authors declare that the research was conducted in the absence of any commercial or financial relationships that could be construed as a potential conflict of interest. The reviewer JL declared a shared affiliation, with one of the author CW to the handling editor at the time of the review.

## Publisher's Note

All claims expressed in this article are solely those of the authors and do not necessarily represent those of their affiliated organizations, or those of the publisher, the editors and the reviewers. Any product that may be evaluated in this article, or claim that may be made by its manufacturer, is not guaranteed or endorsed by the publisher.
