# Supplementary material for: Screening New Blood Indicators for Non-alcoholic Fatty Liver Disease (NAFLD) Diagnosis of Chinese Based on Machine Learning
Source: Front Med (Lausanne). 2022 Jun 9;9:771219. doi: 10.3389/fmed.2022.771219 (PMC9218755; doi:10.3389/fmed.2022.771219)
Supplement: Supplementary file 1 [file Table_1.docx]

**Table S1**. Detailed clinical data for 365 adult individuals

| group | Gender | Age | t-PA | PAI-1 | PAI-1/TPA | BMI | TC | TG | HDL-C | LDL-C | ALT | AST | AST:ALT |
| --- | --- | --- | --- | --- | --- | --- | --- | --- | --- | --- | --- | --- | --- |
| Normal | female | 22 | 4502.732 | 15817.64 | 3.512898 | 20.68 | 4.4 | 0.91 | 1.52 | 2.33 | 13 | 15 | 1.15 |
| Normal | female | 23 | 1956.284 | 14950.67 | 7.642382 | 20.81 | 3.84 | 0.94 | 1.57 | 1.83 | 58 | 47 | 0.81 |
| Normal | female | 23 | 3672.131 | 12603.89 | 3.432309 | 17.46 | 4.04 | 0.68 | 1.71 | 1.9 | 19 | 17 | 0.89 |
| Normal | female | 23 | 4142.077 | 15698.06 | 3.789901 | 19.1 | 3.87 | 0.88 | 1.68 | 1.72 | 9 | 10 | 1.11 |
| Normal | female | 24 | 5344.262 | 18074.74 | 3.382083 | 19.44 | 3.92 | 0.61 | 1.57 | 1.98 | 13 | 17 | 1.31 |
| Normal | female | 24 | 5508.197 | 30959.64 | 5.620649 | 18.71 | 3.58 | 0.97 | 1.6 | 1.47 | 10 | 11 | 1.1 |
| Normal | female | 25 | 4382.514 | 18358.74 | 4.189089 | 18.37 | 3.65 | 0.77 | 1.63 | 1.62 | 11 | 14 | 1.27 |
| Normal | female | 27 | 2743.169 | 13351.27 | 4.867097 | 19.23 | 3.42 | 1.08 | 1.06 | 1.8 | 11 | 15 | 1.36 |
| Normal | female | 27 | 3945.355 | 14606.88 | 3.702298 | 24.56 | 4.5 | 0.86 | 1.29 | 2.49 | 14 | 14 | 1 |
| Normal | female | 27 | 7289.617 | 15384.16 | 2.110421 | 20.06 | 4.01 | 0.64 | 1.73 | 1.78 | 9 | 14 | 1.56 |
| Normal | female | 28 | 1562.842 | 17491.78 | 11.19229 | 18.4 | 3.99 | 0.61 | 1.36 | 2.07 | 8 | 12 | 1.5 |
| Normal | female | 28 | 3278.689 | 12962.63 | 3.953602 | 24.73 | 3.47 | 0.53 | 1.5 | 1.57 | 16 | 19 | 1.19 |
| Normal | female | 28 | 5693.989 | 13605.38 | 2.389429 | 22.19 | 2.54 | 0.88 | 1.07 | 1.07 | 10 | 14 | 1.4 |
| Normal | female | 28 | 6371.585 | 25982.06 | 4.077802 | 21.12 | 4.02 | 0.48 | 1.63 | 1.92 | 9 | 13 | 1.44 |
| Normal | male | 28 | 8655.738 | 22394.62 | 2.587257 | 19.83 | 4.85 | 0.86 | 1.81 | 2.37 | 19 | 18 | 0.95 |
| Normal | male | 29 | 3923.497 | 13799.7 | 3.517194 | 26.53 | 4.94 | 0.57 | 1.9 | 2.31 | 24 | 20 | 0.83 |
| Normal | female | 30 | 6459.016 | 21467.86 | 3.323704 | 16.96 | 3.97 | 1 | 2.05 | 1.39 | 12 | 22 | 1.83 |
| Normal | female | 30 | 7103.825 | 12738.42 | 1.793178 | 22.67 | 3.54 | 0.77 | 1.75 | 1.35 | 9 | 11 | 1.22 |
| Normal | female | 31 | 2808.743 | 25937.22 | 9.234458 | 23.22 | 5.69 | 0.49 | 1.73 | 3.13 | 9 | 16 | 1.78 |
| Normal | male | 31 | 8841.53 | 17820.63 | 2.01556 | 18.48 | 4.29 | 1.03 | 1.49 | 2.12 | 18 | 17 | 0.94 |
| Normal | female | 33 | 2163.934 | 11153.96 | 5.154483 | 19 | 5.41 | 1.79 | 1.41 | 2.91 | 14 | 14 | 1 |
| Normal | female | 34 | 4754.098 | 13695.07 | 2.880687 | 19.96 | 3.44 | 0.73 | 1.45 | 1.44 | 13 | 16 | 1.23 |
| Normal | male | 34 | 5693.989 | 19076.23 | 3.35024 | 24.94 | 4.82 | 1.04 | 1.18 | 2.96 | 23 | 18 | 0.78 |
| Normal | female | 34 | 7300.546 | 17626.31 | 2.414382 | 23.36 | 3.91 | 0.58 | 1.34 | 2.02 | 8 | 10 | 1.25 |
| Normal | female | 35 | 3704.918 | 14517.19 | 3.918357 | 20.98 | 3.66 | 1.03 | 1.19 | 1.77 | 9 | 14 | 1.56 |
| Normal | female | 35 | 4437.158 | 12738.42 | 2.870851 | 24.91 | 3.92 | 0.51 | 1.48 | 1.87 | 17 | 13 | 0.76 |
| Normal | female | 35 | 6251.366 | 13605.38 | 2.176385 | 22.35 | 3.97 | 0.8 | 1.54 | 1.97 | 5 | 13 | 2.6 |
| Normal | female | 36 | 2677.596 | 12648.73 | 4.723913 | 21.22 | 4.41 | 0.4 | 2.15 | 1.82 | 35 | 29 | 0.83 |
| Normal | male | 36 | 5540.984 | 13650.22 | 2.463501 | 28.08 | 4.61 | 0.94 | 1.68 | 2.25 | 16 | 19 | 1.19 |
| Normal | female | 36 | 8808.743 | 13097.16 | 1.486836 | 21.38 | 7.86 | 1.68 | 1.46 | 4.96 | 9 | 15 | 1.67 |
| Normal | female | 36 | 9092.896 | 20750.37 | 2.282042 | 22.34 | 3.35 | 0.51 | 1.3 | 1.53 | 15 | 18 | 1.2 |
| Normal | female | 37 | 4863.388 | 16579.97 | 3.40914 | 22.52 | 3.55 | 0.83 | 1.65 | 1.45 | 13 | 13 | 1 |
| Normal | male | 37 | 6098.361 | 13979.07 | 2.292267 | 22.43 | 5.01 | 1.13 | 1.66 | 2.61 | 25 | 16 | 0.64 |
| Normal | female | 37 | 6797.814 | 12439.46 | 1.829921 | 19.88 | 3.14 | 0.53 | 1.31 | 1.39 | 10 | 12 | 1.2 |
| Normal | male | 37 | 7617.486 | 14248.13 | 1.87045 | 27.31 | 4.82 | 2 | 1.19 | 2.67 | 23 | 16 | 0.7 |
| Normal | female | 37 | 9431.694 | 21961.14 | 2.328441 | 21.79 | 3.68 | 1.01 | 1.26 | 1.84 | 11 | 15 | 1.36 |
| Normal | female | 37 | 9825.137 | 14666.67 | 1.49277 | 24.36 | 4.68 | 1.71 | 1.15 | 2.42 | 30 | 24 | 0.8 |
| Normal | female | 38 | 655.7377 | 12678.62 | 19.3349 | 21.96 | 3.39 | 0.5 | 1.63 | 1.31 | 13 | 11 | 0.85 |
| Normal | female | 38 | 2841.53 | 14427.5 | 5.07737 | 22.88 | 3.35 | 0.73 | 1.49 | 1.36 | 8 | 13 | 1.63 |
| Normal | male | 38 | 9683.06 | 13754.86 | 1.420508 | 19.48 | 4.67 | 1.34 | 1.57 | 2.35 | 26 | 17 | 0.65 |
| Normal | female | 39 | 4371.585 | 16819.13 | 3.847376 | 24.49 | 4.31 | 0.87 | 1.35 | 2.27 | 16 | 19 | 1.19 |
| Normal | female | 39 | 4972.678 | 19001.49 | 3.821178 | 22.09 | 3.77 | 0.38 | 1.82 | 1.51 | 21 | 16 | 0.76 |
| Normal | female | 39 | 6120.219 | 16938.71 | 2.767664 | 22.89 | 3.67 | 0.62 | 1.52 | 1.76 | 15 | 18 | 1.2 |
| Normal | female | 39 | 6513.661 | 16624.81 | 2.552299 | 18.86 | 3.75 | 0.4 | 1.83 | 1.59 | 11 | 14 | 1.27 |
| Normal | male | 39 | 13923.5 | 13426.01 | 0.96427 | 20.13 | 4.82 | 0.84 | 1.29 | 2.86 | 16 | 14 | 0.88 |
| Normal | female | 40 | 2546.448 | 16445.44 | 6.458188 | 20.08 | 3.31 | 0.65 | 1.83 | 1.19 | 23 | 18 | 0.78 |
| Normal | female | 40 | 2622.951 | 14098.65 | 5.37511 | 19.62 | 4.36 | 0.73 | 1.54 | 2.21 | 12 | 15 | 1.25 |
| Normal | female | 40 | 3267.76 | 17237.67 | 5.275072 | 22.33 | 3.08 | 0.54 | 1.36 | 1.33 | 15 | 17 | 1.13 |
| Normal | female | 40 | 5103.825 | 16669.66 | 3.266111 | 22.19 | 3.68 | 1.31 | 0.78 | 2.06 | 16 | 18 | 1.13 |
| Normal | female | 40 | 6295.082 | 16923.77 | 2.688411 | 21.74 | 3.69 | 1.84 | 1.03 | 1.2 | 10 | 12 | 1.2 |
| Normal | female | 40 | 7639.344 | 12439.46 | 1.628341 | 23.55 | 4.98 | 1.11 | 1.42 | 2.75 | 16 | 16 | 1 |
| Normal | female | 40 | 9311.475 | 10197.31 | 1.095134 | 23.16 | 4.58 | 3.35 | 1.1 | 2.22 | 16 | 14 | 0.88 |
| Normal | female | 40 | 11715.85 | 13455.9 | 1.148521 | 20.73 | 3.87 | 0.85 | 1.54 | 1.83 | 11 | 13 | 1.18 |
| Normal | female | 41 | 4786.885 | 13754.86 | 2.873447 | 21.03 | 4.13 | 0.7 | 1.39 | 2.12 | 12 | 9 | 0.75 |
| Normal | female | 41 | 7344.262 | 19554.56 | 2.662563 | 21.11 | 3.95 | 0.99 | 1.42 | 1.84 | 10 | 13 | 1.3 |
| Normal | female | 42 | 3333.333 | 20541.11 | 6.162334 | 22.08 | 5.34 | 0.69 | 1.88 | 2.7 | 32 | 26 | 0.81 |
| Normal | female | 42 | 6961.749 | 19748.88 | 2.83677 | 20.08 | 5.13 | 1.23 | 1.4 | 2.88 | 9 | 11 | 1.22 |
| Normal | male | 42 | 8163.934 | 14053.81 | 1.721451 | 24.43 | 3.91 | 2.41 | 0.8 | 1.68 | 16 | 17 | 1.06 |
| Normal | male | 42 | 8535.519 | 16804.19 | 1.968737 | 22.59 | 4.53 | 0.96 | 1.49 | 2.41 | 16 | 19 | 1.19 |
| Normal | female | 42 | 8786.885 | 18134.53 | 2.063818 | 20.21 | 4.06 | 0.93 | 1.22 | 2.3 | 12 | 14 | 1.17 |
| Normal | female | 43 | 3639.344 | 12289.99 | 3.376979 | 16.96 | 3.26 | 0.73 | 1.79 | 1.14 | 13 | 16 | 1.23 |
| Normal | male | 44 | 10010.93 | 15219.73 | 1.520311 | 19.31 | 4.1 | 1.62 | 1.11 | 2.09 | 16 | 18 | 1.13 |
| Normal | male | 44 | 12131.15 | 13934.23 | 1.148632 | 28.08 | 5.52 | 2.46 | 1.17 | 2.82 | 24 | 21 | 0.88 |
| Normal | female | 44 | 12327.87 | 23515.7 | 1.907523 | 32.36 | 5.1 | 1.07 | 1.43 | 2.93 | 11 | 16 | 1.45 |
| Normal | male | 44 | 13650.27 | 12215.25 | 0.894872 | 19.74 | 5.38 | 2.32 | 1.72 | 2.68 | 24 | 21 | 0.88 |
| Normal | female | 45 | 2120.219 | 18702.54 | 8.821042 | 23.1 | 4.16 | 0.54 | 1.83 | 1.73 | 9 | 14 | 1.56 |
| Normal | female | 45 | 5825.137 | 15010.46 | 2.576842 | 23.82 | 6.05 | 1.03 | 1.04 | 3.89 | 8 | 14 | 1.75 |
| Normal | female | 46 | 1825.137 | 15204.78 | 8.330761 | 25.89 | 4.21 | 0.45 | 1.82 | 1.93 | 12 | 14 | 1.17 |
| Normal | female | 46 | 3322.404 | 20047.83 | 6.034134 | 22.91 | 4.54 | 0.5 | 1.78 | 2.31 | 10 | 15 | 1.5 |
| Normal | female | 46 | 5956.284 | 14741.41 | 2.474934 | 22.81 | 4.59 | 0.97 | 1.44 | 2.33 | 11 | 16 | 1.45 |
| Normal | female | 46 | 7704.918 | 16550.07 | 2.147988 | 22.82 | 5.52 | 1.37 | 1.67 | 2.87 | 22 | 18 | 0.82 |
| Normal | female | 46 | 10830.6 | 19121.08 | 1.765468 | 27.35 | 4.79 | 1.24 | 1.33 | 2.46 | 17 | 19 | 1.12 |
| Normal | male | 46 | 17846.99 | 25100.15 | 1.406408 | 23.2 | 3.98 | 2.23 | 0.8 | 2.18 | 13 | 15 | 1.15 |
| Normal | female | 47 | 6273.224 | 11886.4 | 1.894783 | 24.91 | 4.24 | 0.72 | 1.85 | 1.75 | 14 | 16 | 1.14 |
| Normal | female | 49 | 1158.47 | 17895.37 | 15.44742 | 31.96 | 4.54 | 0.78 | 1.53 | 2.24 | 18 | 18 | 1 |
| Normal | female | 49 | 4513.661 | 12364.72 | 2.739399 | 18.38 | 4.48 | 0.91 | 1.79 | 2.22 | 11 | 17 | 1.55 |
| Normal | male | 49 | 10721.31 | 13710.01 | 1.278763 | 19.62 | 4.2 | 0.99 | 1.34 | 2.3 | 49 | 42 | 0.86 |
| Normal | female | 49 | 10765.03 | 18896.86 | 1.755393 | 28.22 | 4.06 | 1.7 | 1.19 | 1.92 | 12 | 14 | 1.17 |
| Normal | female | 50 | 1748.634 | 12454.41 | 7.122365 | 20.25 | 3.59 | 0.62 | 1.45 | 1.62 | 16 | 15 | 0.94 |
| Normal | female | 50 | 7748.634 | 12050.82 | 1.555219 | 22.88 | 4.69 | 1.59 | 1.71 | 1.97 | 16 | 20 | 1.25 |
| Normal | female | 50 | 7770.492 | 19913.3 | 2.562682 | 19.65 | 4.03 | 0.81 | 1.43 | 2.08 | 9 | 15 | 1.67 |
| Normal | female | 50 | 9628.415 | 16460.39 | 1.709564 | 24.65 | 4.65 | 1.01 | 1.59 | 2.27 | 20 | 14 | 0.7 |
| Normal | female | 51 | 3584.699 | 12304.93 | 3.432626 | 20.45 | 3.89 | 0.77 | 1.28 | 1.9 | 18 | 22 | 1.22 |
| Normal | female | 51 | 7071.038 | 16938.71 | 2.395505 | 27.8 | 5.39 | 1.5 | 1.33 | 3.3 | 15 | 15 | 1 |
| Normal | female | 51 | 8163.934 | 19315.4 | 2.365943 | 19.66 | 4.96 | 0.37 | 1.74 | 2.53 | 20 | 19 | 0.95 |
| Normal | female | 52 | 3300.546 | 24203.29 | 7.333117 | 25.71 | 4.48 | 0.71 | 1.64 | 2.29 | 15 | 25 | 1.67 |
| Normal | female | 52 | 4295.082 | 33082.21 | 7.702347 | 16.96 | 4.23 | 0.95 | 1.57 | 1.93 | 30 | 32 | 1.07 |
| Normal | female | 52 | 7398.907 | 20137.52 | 2.721688 | 23.89 | 5 | 0.97 | 1.48 | 2.65 | 16 | 22 | 1.38 |
| Normal | male | 52 | 13890.71 | 23291.48 | 1.676767 | 24.99 | 3.72 | 1.04 | 0.94 | 2.13 | 19 | 17 | 0.89 |
| Normal | male | 53 | 4786.885 | 16550.07 | 3.457378 | 21.15 | 4.12 | 2.49 | 0.81 | 2.02 | 11 | 13 | 1.18 |
| Normal | female | 53 | 5136.612 | 14203.29 | 2.765109 | 21.28 | 4.19 | 0.96 | 1.24 | 2.25 | 10 | 14 | 1.4 |
| Normal | female | 53 | 11224.04 | 16146.49 | 1.438563 | 22.14 | 5.65 | 0.7 | 1.78 | 3.11 | 13 | 19 | 1.46 |
| Normal | female | 54 | 1803.279 | 13216.74 | 7.329282 | 22 | 5.44 | 0.59 | 1.58 | 3.18 | 28 | 33 | 1.18 |
| Normal | female | 54 | 4939.891 | 11153.96 | 2.257936 | 20.81 | 4.51 | 1.51 | 1.64 | 1.97 | 13 | 15 | 1.15 |
| Normal | female | 54 | 5978.142 | 15832.59 | 2.648413 | 21.15 | 4.74 | 1.34 | 1.18 | 2.59 | 8 | 15 | 1.88 |
| Normal | female | 55 | 3923.497 | 33127.06 | 8.443248 | 20.82 | 5.26 | 0.85 | 1.74 | 2.73 | 15 | 15 | 1 |
| Normal | female | 56 | 4830.601 | 12125.56 | 2.510156 | 26.94 | 6.73 | 3.14 | 1.28 | 3.35 | 16 | 16 | 1 |
| Normal | male | 56 | 8021.858 | 14173.39 | 1.766846 | 24.73 | 2.63 | 1.69 | 0.79 | 1.11 | 26 | 25 | 0.96 |
| Normal | male | 58 | 12557.38 | 15115.1 | 1.203683 | 27.77 | 3.98 | 0.9 | 1.32 | 1.96 | 15 | 23 | 1.53 |
| NAFLD | female | 26 | 7934.426 | 25085.2 | 3.161565 | 25.35 | 3.35 | 1.28 | 0.98 | 1.81 | 9 | 13 | 1.44 |
| NAFLD | male | 26 | 9118.579 | 34621.82 | 3.796844 | 35.79 | 3.74 | 0.84 | 1.24 | 1.95 | 24 | 34 | 1.42 |
| NAFLD | female | 26 | 9562.842 | 34023.92 | 3.55793 | 28.89 | 3.36 | 1.21 | 1.16 | 1.72 | 17 | 17 | 1 |
| NAFLD | female | 26 | 13595.63 | 20750.37 | 1.526253 | 28.34 | 4.29 | 1.93 |  |  | 47 | 33 | 0.7 |
| NAFLD | female | 27 | 5656.284 | 33739.91 | 5.965031 | 25.59 | 4.59 | 1.49 | 1.36 | 2.69 | 15 | 16 | 1.07 |
| NAFLD | male | 27 | 9715.847 | 35563.53 | 3.660363 | 22.73 | 4.27 | 1.55 | 1 | 2.33 | 12 | 15 | 1.25 |
| NAFLD | male | 27 | 11092.9 | 43500.75 | 3.921495 | 34.18 | 5.36 | 1.64 | 1.32 | 3.12 | 47 | 26 | 0.55 |
| NAFLD | male | 27 | 15770.49 | 34980.57 | 2.218103 | 30.16 | 5.14 | 3.18 | 0.95 | 2.74 | 166 | 66 | 0.4 |
| NAFLD | male | 28 | 4844.809 | 34188.34 | 7.056695 | 34.42 | 5.32 | 2.49 | 1.11 | 3.19 | 42 | 22 | 0.52 |
| NAFLD | male | 28 | 8343.169 | 40212.26 | 4.819783 | 23.46 | 4.13 | 2.73 | 1.09 | 1.94 | 38 | 26 | 0.68 |
| NAFLD | male | 28 | 9519.126 | 31721.97 | 3.332446 | 27.29 | 5.08 | 1.81 | 0.97 | 3.1 | 65 | 30 | 0.46 |
| NAFLD | male | 28 | 9573.77 | 30899.85 | 3.227553 | 26.74 | 5.08 | 2.51 | 1.05 | 2.65 | 163 | 61 | 0.37 |
| NAFLD | male | 28 | 13344.26 | 23769.81 | 1.781276 | 30.5 | 5.98 | 2.8 | 0.71 | 3.4 | 42 | 23 | 0.55 |
| NAFLD | male | 29 | 4910.929 | 31124.07 | 6.337715 | 26.44 | 4.19 | 1.37 | 1.34 | 2.21 | 35 | 21 | 0.6 |
| NAFLD | male | 29 | 7056.831 | 45623.32 | 6.465129 | 32.7 | 4.62 | 1.51 | 1.03 | 2.88 | 241 | 91 | 0.38 |
| NAFLD | male | 29 | 11715.85 | 24681.61 | 2.106685 | 35.22 | 7.02 | 1.61 | 0.85 | 4.84 | 232 | 90 | 0.39 |
| NAFLD | female | 30 | 4219.672 | 39973.09 | 9.473033 | 26.13 | 4.48 | 1.29 | 0.93 | 2.91 | 23 | 19 | 0.83 |
| NAFLD | male | 30 | 4644.809 | 27013.45 | 5.815837 | 30.07 | 5.35 | 1.26 | 0.86 | 3.64 | 32 | 20 | 0.63 |
| NAFLD | male | 30 | 5524.044 | 32095.67 | 5.810176 | 31.69 | 6.23 | 5.79 | 0.95 | 2.68 | 153 | 86 | 0.56 |
| NAFLD | male | 30 | 8415.301 | 43829.6 | 5.208322 | 27.61 | 4.71 | 1.76 | 1.13 | 2.58 | 29 | 19 | 0.66 |
| NAFLD | male | 30 | 13650.27 | 27118.09 | 1.986634 | 28.21 | 5.26 | 4.38 | 0.9 | 2.28 | 38 | 30 | 0.79 |
| NAFLD | male | 30 | 22786.89 | 39584.45 | 1.737159 | 29.48 | 5.64 | 1.76 | 1.21 | 3.21 | 216 | 83 | 0.38 |
| NAFLD | male | 31 | 5486.339 | 37162.93 | 6.773721 | 24.76 | 4.4 | 0.93 | 1.08 | 2.74 | 94 | 33 | 0.35 |
| NAFLD | male | 31 | 6087.432 | 44666.67 | 7.337523 | 23.61 | 4.66 | 2.82 | 1 | 2.22 | 31 | 24 | 0.77 |
| NAFLD | male | 31 | 11683.06 | 34427.5 | 2.946788 | 24.71 | 4.42 | 2.3 | 1.03 | 2.19 | 29 | 25 | 0.86 |
| NAFLD | female | 31 | 12480.87 | 38298.95 | 3.068612 | 22.35 | 5.4 | 1.5 | 1.25 | 3.04 | 14 | 13 | 0.93 |
| NAFLD | male | 32 | 2127.869 | 45130.04 | 21.20903 | 23.29 | 4.66 | 0.96 | 1.48 | 2.6 | 14 | 16 | 1.14 |
| NAFLD | male | 32 | 3155.738 | 31841.55 | 10.09005 | 26.93 | 4.88 | 2.95 | 1.3 | 2.25 | 11 | 20 | 1.82 |
| NAFLD | male | 32 | 3594.536 | 33216.74 | 9.240898 | 27.89 | 5.32 | 0.76 | 1.47 | 3.09 | 22 | 19 | 0.86 |
| NAFLD | male | 32 | 5061.202 | 42753.36 | 8.447274 | 27.23 | 4.53 | 1.86 | 1.17 | 2.64 | 25 | 26 | 1.04 |
| NAFLD | male | 32 | 5092.896 | 19763.83 | 3.880666 | 28.15 | 4.07 | 1.32 | 0.85 | 2.41 | 16 | 15 | 0.94 |
| NAFLD | male | 32 | 5211.475 | 41677.13 | 7.997185 | 28.11 | 5.18 | 1.49 | 1.18 | 3.15 | 40 | 19 | 0.47 |
| NAFLD | female | 32 | 7375.41 | 41273.54 | 5.596101 | 21.05 | 3.67 | 1.13 | 1.13 | 1.87 | 47 | 35 | 0.74 |
| NAFLD | male | 32 | 8612.022 | 30899.85 | 3.58799 | 26.62 | 4.12 | 1 | 1.09 | 2.38 | 40 | 21 | 0.52 |
| NAFLD | male | 32 | 11289.62 | 44696.56 | 3.959085 | 32.69 | 4 | 2.21 | 1.03 | 2.18 | 52 | 28 | 0.54 |
| NAFLD | male | 32 | 12000 | 34681.61 | 2.890134 | 23.86 | 3.98 | 2.88 | 0.86 | 1.92 | 20 | 16 | 0.8 |
| NAFLD | female | 33 | 6896.175 | 22349.78 | 3.240895 | 28.06 | 4.04 | 2.54 | 0.85 | 1.98 | 20 | 17 | 0.85 |
| NAFLD | male | 33 | 8076.503 | 16221.23 | 2.008447 | 22.19 | 4.29 | 2.23 | 1 | 2.33 | 26 | 17 | 0.65 |
| NAFLD | male | 33 | 9475.41 | 24965.62 | 2.63478 | 30.94 | 5.24 | 0.95 | 1.28 | 3.12 | 45 | 23 | 0.51 |
| NAFLD | male | 33 | 9934.426 | 23665.17 | 2.382138 | 21.63 | 5.94 | 1.09 | 1.32 | 3.61 | 20 | 18 | 0.9 |
| NAFLD | male | 33 | 10480.87 | 19031.39 | 1.815822 | 21.48 | 5.12 | 2.48 | 1.12 | 2.59 | 40 | 24 | 0.6 |
| NAFLD | male | 33 | 10633.88 | 23665.17 | 2.22545 | 26.79 | 4.86 | 1.24 | 1.07 | 3.13 | 20 | 15 | 0.75 |
| NAFLD | male | 33 | 11213.11 | 31751.87 | 2.831674 | 25.68 | 5.1 | 2.4 | 0.86 | 2.52 | 31 | 20 | 0.65 |
| NAFLD | male | 33 | 15945.36 | 32693.57 | 2.05035 | 26.98 | 5.06 | 1.63 | 0.73 | 3.44 | 44 | 27 | 0.61 |
| NAFLD | female | 34 | 3871.038 | 45055.31 | 11.63908 | 24.72 | 4.87 | 1.29 | 1.32 | 2.64 | 6 | 12 | 2 |
| NAFLD | male | 34 | 4802.732 | 37207.77 | 7.747209 | 33.58 | 4.33 | 1.69 | 1.02 | 2.76 | 26 | 18 | 0.69 |
| NAFLD | male | 34 | 4953.005 | 35533.63 | 7.174156 | 27.02 | 5.78 | 2.3 | 0.88 | 3.35 | 35 | 18 | 0.51 |
| NAFLD | male | 34 | 6155.191 | 35369.21 | 5.746241 | 26.37 | 5.55 | 1.58 | 1.17 | 3.48 | 68 | 39 | 0.57 |
| NAFLD | male | 34 | 6557.377 | 21632.29 | 3.298924 | 26.62 | 6.12 | 1.09 | 1.06 | 3.98 | 19 | 15 | 0.79 |
| NAFLD | male | 34 | 8983.607 | 23665.17 | 2.634261 | 25.53 | 3.43 | 1.09 | 1.01 | 1.77 | 56 | 30 | 0.54 |
| NAFLD | male | 34 | 16721.31 | 33680.12 | 2.014203 | 23.85 | 4.68 | 1.95 | 0.98 | 2.41 | 39 | 23 | 0.59 |
| NAFLD | male | 35 | 5379.781 | 34995.52 | 6.505008 | 25.9 | 4.32 | 1.37 | 1.26 | 2.64 | 23 | 18 | 0.78 |
| NAFLD | male | 35 | 10928.96 | 33739.91 | 3.087202 | 23.24 | 5.57 | 2.37 | 1.21 | 2.68 | 18 | 19 | 1.06 |
| NAFLD | male | 35 | 11114.75 | 24292.97 | 2.185651 | 28.79 | 4.32 | 2.57 | 0.87 | 2.05 | 39 | 24 | 0.62 |
| NAFLD | male | 35 | 12251.37 | 32095.67 | 2.619762 | 29.94 | 6.75 | 4.55 | 1.08 | 3.57 | 50 | 34 | 0.68 |
| NAFLD | male | 35 | 13311.48 | 23680.12 | 1.778925 | 24.92 | 5.39 | 5.79 | 1.02 | 2 | 27 | 21 | 0.78 |
| NAFLD | male | 35 | 13803.28 | 44352.77 | 3.213205 | 31.19 | 6.19 | 2.06 | 1.38 | 3.52 | 73 | 39 | 0.53 |
| NAFLD | female | 36 | 4934.973 | 37476.83 | 7.594131 | 25.44 | 3.98 | 0.98 | 1.16 | 2.2 | 15 | 18 | 1.2 |
| NAFLD | male | 36 | 8306.011 | 28866.97 | 3.475431 | 25.9 | 5.65 | 0.81 | 1.53 | 3.61 | 44 | 22 | 0.5 |
| NAFLD | male | 36 | 8619.672 | 33799.7 | 3.921228 | 28.77 | 4.15 | 1.06 | 0.86 | 2.66 | 16 | 16 | 1 |
| NAFLD | male | 36 | 15147.54 | 21632.29 | 1.428106 | 28.49 | 4.13 | 6.69 | 0.59 | 1.39 | 63 | 27 | 0.43 |
| NAFLD | female | 37 | 3810.929 | 35563.53 | 9.331984 | 25.71 | 6.06 | 1.26 | 1.33 | 3.76 | 8 | 16 | 2 |
| NAFLD | male | 37 | 4916.94 | 45548.58 | 9.263603 | 28.94 | 4.08 | 2.19 | 1.1 | 2.18 | 31 | 19 | 0.61 |
| NAFLD | male | 37 | 7014.754 | 30840.06 | 4.396456 | 27.8 | 4.29 | 1.98 | 1.14 | 2.34 | 35 | 20 | 0.57 |
| NAFLD | male | 37 | 7759.563 | 23530.64 | 3.03247 | 24.29 | 4.9 | 2.16 | 0.98 | 2.85 | 19 | 15 | 0.79 |
| NAFLD | male | 37 | 10360.66 | 40002.99 | 3.861046 | 26.66 | 3.73 | 3.42 | 0.89 | 1.65 | 59 | 29 | 0.49 |
| NAFLD | male | 37 | 10459.02 | 24442.45 | 2.336973 | 28.71 | 4.69 | 1.43 | 1.1 | 2.86 | 42 | 23 | 0.55 |
| NAFLD | male | 37 | 16633.88 | 23829.6 | 1.432594 | 30.21 | 6.68 | 7.88 | 0.92 | 1.94 | 29 | 18 | 0.62 |
| NAFLD | male | 38 | 4375.956 | 35832.59 | 8.188517 | 23.46 | 3.69 | 1.4 | 0.77 | 2.28 | 23 | 17 | 0.74 |
| NAFLD | female | 38 | 6089.071 | 35787.74 | 5.877373 | 27.89 | 4.83 | 1.1 | 1.35 | 2.92 | 11 | 13 | 1.18 |
| NAFLD | male | 38 | 7027.322 | 20660.69 | 2.940052 | 27.33 | 4.08 | 0.99 | 1.14 | 2.36 | 25 | 21 | 0.84 |
| NAFLD | female | 38 | 7681.967 | 38523.17 | 5.014753 | 30.4 | 4.92 | 1.12 | 1 | 3.19 | 37 | 24 | 0.65 |
| NAFLD | male | 38 | 10841.53 | 39898.36 | 3.680141 | 25.8 | 4.72 | 1.39 | 1.03 | 2.77 | 30 | 25 | 0.83 |
| NAFLD | male | 38 | 11027.32 | 30511.21 | 2.766874 | 24.19 | 5.71 | 2.66 | 0.84 | 3.32 | 47 | 28 | 0.6 |
| NAFLD | male | 38 | 13661.2 | 19988.04 | 1.463125 | 24.49 | 4.01 | 1.48 | 0.93 | 2.1 | 25 | 20 | 0.8 |
| NAFLD | male | 38 | 14229.51 | 22813.15 | 1.603228 | 25.74 | 4.56 | 3.28 | 0.99 | 2.42 | 39 | 29 | 0.74 |
| NAFLD | female | 39 | 5247.541 | 30496.26 | 5.811533 | 22.2 | 5.33 | 1.28 | 1.41 | 3.04 | 22 | 21 | 0.95 |
| NAFLD | female | 39 | 5566.12 | 34771.3 | 6.246955 | 21.87 | 4.41 | 2.46 | 1.12 | 2.07 | 13 | 15 | 1.15 |
| NAFLD | male | 39 | 5879.781 | 20227.2 | 3.440128 | 29.24 | 5.65 | 3.77 | 0.99 | 2.96 | 37 | 21 | 0.57 |
| NAFLD | male | 39 | 6545.902 | 41826.61 | 6.38974 | 23.68 | 4.52 | 1.71 | 1.33 | 2.35 | 21 | 23 | 1.1 |
| NAFLD | female | 39 | 9169.399 | 35802.69 | 3.904584 | 23.41 | 5.03 | 1.47 | 1.33 | 2.77 | 39 | 20 | 0.51 |
| NAFLD | male | 39 | 10065.57 | 22110.61 | 2.196658 | 23.55 | 4.09 | 1.01 | 1.29 | 2.19 | 13 | 21 | 1.62 |
| NAFLD | male | 39 | 12863.39 | 34263.08 | 2.663612 | 24.15 | 5.31 | 1.39 | 1.26 | 2.98 | 14 | 18 | 1.29 |
| NAFLD | male | 39 | 13497.27 | 36385.65 | 2.695778 | 23.04 | 4.03 | 0.86 | 1.55 | 2.05 | 25 | 28 | 1.12 |
| NAFLD | female | 39 | 13530.05 | 45294.47 | 3.347694 | 26.39 | 3.68 | 0.66 | 0.92 | 2.04 | 11 | 11 | 1 |
| NAFLD | male | 39 | 14153.01 | 23605.38 | 1.66787 | 27.07 | 3.94 | 1.88 | 0.73 | 2.4 | 28 | 18 | 0.64 |
| NAFLD | male | 39 | 14633.88 | 30750.37 | 2.101314 | 30.52 | 6.69 | 3.28 | 1.04 | 3.84 | 21 | 20 | 0.95 |
| NAFLD | male | 39 | 15049.18 | 32544.1 | 2.162516 | 31.51 | 5.51 | 1.09 | 0.89 | 3.53 | 169 | 58 | 0.34 |
| NAFLD | male | 40 | 3737.705 | 24412.56 | 6.53143 | 24.61 | 4.22 | 0.9 | 1.29 | 2.29 | 27 | 22 | 0.81 |
| NAFLD | male | 40 | 5180.328 | 22588.94 | 4.360523 | 28.78 | 4.5 | 2.62 | 0.91 | 1.89 | 32 | 20 | 0.63 |
| NAFLD | male | 40 | 5355.191 | 14651.72 | 2.735985 | 24.81 | 3.36 | 1.69 | 0.76 | 1.41 | 38 | 17 | 0.45 |
| NAFLD | male | 40 | 6858.47 | 36953.66 | 5.388033 | 24.73 | 6.09 | 2.23 | 1.15 | 3.61 | 29 | 21 | 0.72 |
| NAFLD | male | 40 | 9868.852 | 21168.91 | 2.145023 | 24.49 | 4.85 | 2.24 | 0.86 | 2.6 | 44 | 23 | 0.52 |
| NAFLD | male | 40 | 11081.97 | 43127.06 | 3.891642 | 24.24 | 6.08 | 1.14 | 1.66 | 3.49 | 31 | 26 | 0.84 |
| NAFLD | male | 40 | 11617.49 | 22962.63 | 1.976557 | 28.68 | 4.49 | 2.17 | 0.82 | 2.52 | 35 | 28 | 0.8 |
| NAFLD | male | 40 | 11748.63 | 23231.69 | 1.977396 | 54.05 | 3.13 | 1.13 | 1.15 | 1.18 | 19 | 18 | 0.95 |
| NAFLD | male | 40 | 12196.72 | 23844.54 | 1.954996 | 27.96 | 4.09 | 3.85 | 0.86 | 1.38 | 43 | 28 | 0.65 |
| NAFLD | male | 40 | 13256.83 | 30615.84 | 2.309439 | 25.52 | 4.44 | 1.34 | 0.98 | 2.7 | 29 | 23 | 0.79 |
| NAFLD | male | 40 | 20174.86 | 17297.46 | 0.857377 | 24.26 | 4.3 | 3.26 | 0.94 | 1.72 | 55 | 30 | 0.55 |
| NAFLD | male | 41 | 4284.153 | 18029.9 | 4.20851 | 30.08 | 5.63 | 2.8 | 0.95 | 3 | 37 | 19 | 0.51 |
| NAFLD | male | 41 | 5620.219 | 33994.02 | 6.048522 | 29.79 | 4.93 | 2.21 | 1.15 | 2.86 | 81 | 42 | 0.52 |
| NAFLD | male | 41 | 5644.262 | 39076.23 | 6.923178 | 26.21 | 5.15 | 2.95 | 1.17 | 2.47 | 21 | 20 | 0.95 |
| NAFLD | male | 41 | 5956.831 | 31378.18 | 5.267596 | 24.99 | 3.28 | 1.05 | 1.06 | 1.79 | 34 | 27 | 0.79 |
| NAFLD | male | 41 | 6251.366 | 31587.44 | 5.052886 | 25.25 | 3.77 | 1.85 | 1.31 | 1.73 | 20 | 18 | 0.9 |
| NAFLD | female | 41 | 6284.153 | 31976.08 | 5.088368 | 23.91 | 3.89 | 0.98 | 1.49 | 1.86 | 15 | 16 | 1.07 |
| NAFLD | male | 41 | 6852.459 | 25130.04 | 3.667302 | 26.93 | 5.02 | 1.01 | 0.94 | 3.2 | 37 | 21 | 0.57 |
| NAFLD | male | 41 | 6852.459 | 23635.28 | 3.449168 | 27.31 | 5.56 | 2.17 | 1.48 | 2.95 | 42 | 29 | 0.69 |
| NAFLD | male | 41 | 7016.393 | 20167.41 | 2.874327 | 25.73 | 4.2 | 2.55 | 1.31 | 1.88 | 51 | 30 | 0.59 |
| NAFLD | male | 41 | 7453.552 | 16026.91 | 2.150238 | 24.85 | 6.54 | 2.32 | 0.85 | 4.12 | 30 | 19 | 0.63 |
| NAFLD | female | 41 | 7994.536 | 41034.38 | 5.132803 | 24.68 | 3.56 | 1.14 | 1.26 | 1.72 | 18 | 16 | 0.89 |
| NAFLD | male | 41 | 8404.372 | 22334.83 | 2.657525 | 31.23 | 3.63 | 1.19 | 0.84 | 2 | 26 | 21 | 0.81 |
| NAFLD | female | 41 | 9060.109 | 39330.34 | 4.341045 | 33.12 | 3.81 | 1.82 | 1.11 | 1.8 | 15 | 16 | 1.07 |
| NAFLD | male | 41 | 9683.06 | 15503.74 | 1.60112 | 23.77 | 5.19 | 1.88 | 1.43 | 2.8 | 31 | 21 | 0.68 |
| NAFLD | male | 41 | 10153.01 | 32155.46 | 3.167086 | 26.25 | 5.25 | 1.59 | 1.02 | 3.25 | 21 | 15 | 0.71 |
| NAFLD | male | 41 | 10644.81 | 35324.36 | 3.318458 | 24.59 | 3.36 | 0.65 | 1.1 | 1.89 | 61 | 32 | 0.52 |
| NAFLD | male | 41 | 11366.12 | 39046.34 | 3.435327 | 27.09 | 4.34 | 1.53 | 1.11 | 2.37 | 30 | 25 | 0.83 |
| NAFLD | female | 41 | 11420.77 | 44726.46 | 3.916239 | 24.96 | 7.45 | 1.72 | 1.08 | 4.94 | 39 | 21 | 0.54 |
| NAFLD | male | 41 | 13377.05 | 21333.33 | 1.594771 | 27.44 | 4.67 | 0.91 | 1.34 | 2.53 | 31 | 27 | 0.87 |
| NAFLD | female | 41 | 13573.77 | 22798.21 | 1.679578 | 21.57 | 4.52 | 0.87 | 1.37 | 2.34 | 15 | 19 | 1.27 |
| NAFLD | male | 41 | 18841.53 | 29524.66 | 1.566999 | 27.8 | 4.5 | 2.36 | 0.8 | 2.69 | 27 | 21 | 0.78 |
| NAFLD | male | 42 | 2316.94 | 21213.75 | 9.155934 | 20.83 | 3.35 | 1.02 | 0.96 | 1.79 | 11 | 17 | 1.55 |
| NAFLD | male | 42 | 3883.06 | 46759.34 | 12.04188 | 27.74 | 3.18 | 1.78 | 0.8 | 1.62 | 12 | 14 | 1.17 |
| NAFLD | male | 42 | 5235.519 | 33097.16 | 6.321658 | 28.57 | 6.14 | 2.3 | 0.97 | 3.75 | 36 | 20 | 0.56 |
| NAFLD | female | 42 | 5457.923 | 38089.69 | 6.978788 | 25.57 | 6.08 | 2.78 | 1.01 | 3.56 | 21 | 21 | 1 |
| NAFLD | male | 42 | 7159.016 | 28418.54 | 3.969615 | 26.69 | 6.42 | 8.2 | 0.8 | 3.09 | 11 | 11 | 1 |
| NAFLD | male | 42 | 8032.787 | 20436.47 | 2.544132 | 32.71 | 4.62 | 1.35 | 0.94 | 2.62 | 47 | 31 | 0.66 |
| NAFLD | male | 42 | 8437.158 | 26415.55 | 3.130859 | 30.01 | 5.48 | 1.96 | 1.04 | 3.28 | 48 | 31 | 0.65 |
| NAFLD | male | 42 | 8885.246 | 21482.81 | 2.417807 | 30.34 | 4.69 | 1.97 | 1.32 | 2.39 | 20 | 14 | 0.7 |
| NAFLD | male | 42 | 9737.705 | 21497.76 | 2.207682 | 25.09 | 3.67 | 1.18 | 0.94 | 2.16 | 36 | 22 | 0.61 |
| NAFLD | male | 42 | 10087.43 | 17730.94 | 1.757726 | 24.94 | 5.77 | 2.11 | 0.98 | 3.33 | 29 | 20 | 0.69 |
| NAFLD | male | 42 | 10557.38 | 22155.46 | 2.098576 | 30.77 | 6.6 | 3.12 | 1.05 | 3.41 | 15 | 24 | 1.6 |
| NAFLD | male | 42 | 13464.48 | 19629.3 | 1.457858 | 25.22 | 4.72 | 1.87 | 1.17 | 2.7 | 35 | 22 | 0.63 |
| NAFLD | male | 42 | 15387.98 | 22484.3 | 1.46116 | 26.07 | 6.91 | 4.54 | 1.06 | 3.1 | 27 | 25 | 0.93 |
| NAFLD | male | 42 | 17540.98 | 19778.77 | 1.127575 | 26.8 | 5.19 | 1.33 | 1.35 | 2.95 | 19 | 14 | 0.74 |
| NAFLD | male | 42 | 19846.99 | 37282.51 | 1.878497 | 25.07 | 5.83 | 5.07 | 1.1 | 2.37 | 16 | 16 | 1 |
| NAFLD | male | 43 | 4972.678 | 26071.75 | 5.243 | 25.27 | 3.64 | 0.51 | 1.41 | 1.74 | 13 | 14 | 1.08 |
| NAFLD | male | 43 | 8874.317 | 23336.32 | 2.629647 | 24.31 | 5.07 | 1.43 | 1.65 | 2.53 | 18 | 15 | 0.83 |
| NAFLD | male | 43 | 10174.86 | 28508.22 | 2.801829 | 26.55 | 6.58 | 1.98 | 1.44 | 3.85 | 31 | 29 | 0.94 |
| NAFLD | male | 43 | 10382.51 | 28777.28 | 2.771707 | 23.93 | 5.37 | 1.01 | 1.27 | 3.15 | 42 | 35 | 0.83 |
| NAFLD | male | 43 | 14448.09 | 42947.68 | 2.972551 | 32.02 | 4.78 | 1.78 | 1.09 | 2.74 | 46 | 29 | 0.63 |
| NAFLD | male | 43 | 14568.31 | 25219.73 | 1.731136 | 26.85 | 5.13 | 1.51 | 1.12 | 3 | 22 | 17 | 0.77 |
| NAFLD | male | 43 | 19792.35 | 23650.22 | 1.194917 | 30.08 | 3.54 | 1.95 | 0.86 | 1.83 | 41 | 42 | 1.02 |
| NAFLD | male | 43 | 22994.54 | 40301.94 | 1.752674 | 32.28 | 4.51 | 2.03 | 1.17 | 2.41 | 24 | 21 | 0.88 |
| NAFLD | male | 44 | 1202.19 | 29210.76 | 24.29796 | 27.37 | 5.53 | 1.13 | 1.17 | 3.5 | 39 | 27 | 0.69 |
| NAFLD | male | 44 | 4285.792 | 38418.54 | 8.964163 | 23.43 | 4.29 | 2 | 1.16 | 2.22 | 46 | 22 | 0.48 |
| NAFLD | male | 44 | 5224.044 | 22095.67 | 4.22961 | 25.12 | 2.9 | 1.07 | 0.97 | 1.12 | 28 | 27 | 0.96 |
| NAFLD | male | 44 | 6557.923 | 39868.46 | 6.079434 | 32.3 | 4.43 | 0.67 | 1.56 | 2.44 | 30 | 29 | 0.97 |
| NAFLD | male | 44 | 8786.885 | 38792.23 | 4.414787 | 31.37 | 3.66 | 1.08 | 0.84 | 2.03 | 39 | 25 | 0.64 |
| NAFLD | male | 44 | 10535.52 | 23560.54 | 2.236296 | 28.22 | 6.66 | 3.56 | 1.04 | 3.05 | 16 | 17 | 1.06 |
| NAFLD | male | 45 | 4412.022 | 31542.6 | 7.149239 | 27.09 | 4.03 | 1.41 | 0.92 | 2.38 | 23 | 22 | 0.96 |
| NAFLD | male | 45 | 5205.464 | 27327.35 | 5.249743 | 27.8 | 4.62 | 1.02 | 1.42 | 2.53 | 26 | 27 | 1.04 |
| NAFLD | male | 45 | 6579.235 | 22768.31 | 3.460632 | 24.25 | 4.12 | 1.37 | 1.16 | 2.1 | 32 | 16 | 0.5 |
| NAFLD | male | 45 | 9060.109 | 20032.88 | 2.211108 | 22.09 | 6.19 | 1.67 | 1.8 | 3.28 | 33 | 27 | 0.82 |
| NAFLD | male | 45 | 16043.72 | 35847.53 | 2.234365 | 25.78 | 3.45 | 1.35 | 1.11 | 1.75 | 16 | 15 | 0.94 |
| NAFLD | female | 46 | 3103.825 | 25548.58 | 8.231321 | 26.53 | 4.76 | 1.63 | 0.97 | 2.66 | 19 | 17 | 0.89 |
| NAFLD | male | 46 | 8433.333 | 36460.39 | 4.323367 | 27.96 | 4.38 | 0.64 | 1.09 | 2.79 | 22 | 13 | 0.59 |
| NAFLD | female | 46 | 8448.087 | 44816.14 | 5.304886 | 28.2 | 4.14 | 1.37 | 1.43 | 2.03 | 22 | 23 | 1.05 |
| NAFLD | male | 46 | 12677.6 | 34098.65 | 2.689677 | 25.91 | 5.22 | 2.35 | 1.14 | 2.95 | 23 | 21 | 0.91 |
| NAFLD | male | 46 | 15978.14 | 37551.57 | 2.350184 | 27.36 | 3.8 | 1.54 | 0.99 | 1.93 | 69 | 36 | 0.52 |
| NAFLD | female | 46 | 16404.37 | 38358.74 | 2.338324 | 31.25 | 4.06 | 1.46 | 1.17 | 2.11 | 19 | 12 | 0.63 |
| NAFLD | male | 46 | 17540.98 | 35653.21 | 2.032567 | 20.57 | 2.91 | 0.87 | 1.57 | 0.62 | 16 | 17 | 1.06 |
| NAFLD | female | 46 | 19879.78 | 36490.28 | 1.835547 | 26.36 | 5.12 | 1.54 | 1.56 | 2.79 | 20 | 27 | 1.35 |
| NAFLD | male | 46 | 22295.08 | 26475.34 | 1.187497 | 25.49 | 4.74 | 2.27 | 1.02 | 2.34 | 47 | 29 | 0.62 |
| NAFLD | male | 47 | 7453.552 | 26385.65 | 3.54001 | 25.59 | 4.5 | 0.99 | 1.26 | 2.44 | 22 | 22 | 1 |
| NAFLD | male | 47 | 8481.421 | 31079.22 | 3.664388 | 27.95 | 4.21 | 2.01 | 1.01 | 2.24 | 21 | 20 | 0.95 |
| NAFLD | male | 47 | 8710.383 | 27805.68 | 3.192245 | 31.73 | 4.83 | 2.2 | 0.9 | 2.85 | 21 | 19 | 0.9 |
| NAFLD | male | 47 | 10251.37 | 30212.26 | 2.947144 | 23.26 | 9.98 | 1.59 | 1 | 7.4 | 22 | 18 | 0.82 |
| NAFLD | male | 47 | 10852.46 | 34621.82 | 3.190228 | 26.33 | 5.18 | 0.97 | 1.34 | 3.13 | 21 | 16 | 0.76 |
| NAFLD | male | 47 | 14076.5 | 36146.49 | 2.567861 | 26.91 | 4.49 | 1.92 | 0.77 | 2.62 | 21 | 20 | 0.95 |
| NAFLD | male | 47 | 17584.7 | 20825.11 | 1.184274 | 30.2 | 4.5 | 5.4 | 0.76 | 1.57 | 134 | 57 | 0.43 |
| NAFLD | female | 48 | 3720.765 | 29928.25 | 8.043574 | 26.22 | 4.81 | 0.71 | 1.28 | 2.89 | 37 | 31 | 0.84 |
| NAFLD | male | 48 | 7357.377 | 35189.84 | 4.782933 | 22.56 | 4.59 | 1.29 | 1.01 | 2.7 | 70 | 34 | 0.49 |
| NAFLD | male | 48 | 9103.825 | 21677.13 | 2.381101 | 31.55 | 2.35 | 1.06 | 0.86 | 1.08 | 25 | 18 | 0.72 |
| NAFLD | male | 48 | 11180.33 | 36176.38 | 3.235717 | 22.92 | 4.65 | 4.26 | 0.95 | 1.99 | 14 | 9 | 0.64 |
| NAFLD | male | 48 | 12256.28 | 35518.68 | 2.897998 | 23.29 | 5.81 | 7.52 | 1.05 | 1.67 | 28 | 18 | 0.64 |
| NAFLD | male | 48 | 13016.39 | 44113.6 | 3.389081 | 27.91 | 5.11 | 2.29 | 1.29 | 2.81 | 26 | 20 | 0.77 |
| NAFLD | male | 48 | 13722.95 | 42753.36 | 3.115464 | 27.27 | 4.46 | 4.17 | 1.04 | 1.69 | 21 | 20 | 0.95 |
| NAFLD | female | 48 | 14590.16 | 25294.47 | 1.733666 | 25.25 | 6.86 | 5.21 | 1.19 | 2.77 | 15 | 20 | 1.33 |
| NAFLD | female | 49 | 4922.951 | 29180.87 | 5.927516 | 26.68 | 7.3 | 1.55 | 1.37 | 4.87 | 14 | 14 | 1 |
| NAFLD | female | 49 | 5748.634 | 31946.19 | 5.557179 | 25.95 | 3.57 | 1.12 | 1.25 | 1.81 | 11 | 11 | 1 |
| NAFLD | male | 49 | 6059.016 | 31318.39 | 5.16889 | 26.37 | 5.04 | 1.4 | 1.17 | 3 | 18 | 20 | 1.11 |
| NAFLD | male | 49 | 9300.546 | 20077.73 | 2.158769 | 28.6 | 5.27 | 1.75 | 1.22 | 3.1 | 20 | 21 | 1.05 |
| NAFLD | male | 49 | 9683.06 | 37252.62 | 3.847195 | 24.98 | 4.57 | 1.13 | 1.21 | 2.48 | 27 | 17 | 0.63 |
| NAFLD | male | 49 | 9683.607 | 37043.35 | 3.825367 | 25.59 | 5.47 | 0.89 | 1.63 | 3.19 | 26 | 23 | 0.88 |
| NAFLD | female | 49 | 11180.33 | 27222.72 | 2.434876 | 25.04 | 5.35 | 1.31 | 1.53 | 3.11 | 23 | 18 | 0.78 |
| NAFLD | male | 49 | 11464.48 | 23097.16 | 2.014671 | 24.89 | 5.31 | 2.63 | 1.18 | 2.91 | 35 | 22 | 0.63 |
| NAFLD | female | 49 | 13453.55 | 21318.39 | 1.584592 | 29.31 | 4.96 | 2.13 | 0.94 | 2.76 | 13 | 14 | 1.08 |
| NAFLD | male | 49 | 16382.51 | 33411.06 | 2.039435 | 23.44 | 4.94 | 1.64 | 0.93 | 2.87 | 24 | 16 | 0.67 |
| NAFLD | male | 49 | 18480.87 | 36056.8 | 1.951034 | 29.67 | 6.27 | 2.08 | 1.66 | 2.65 | 20 | 21 | 1.05 |
| NAFLD | male | 50 | 4880.874 | 47118.09 | 9.653617 | 24.46 | 4.85 | 2.05 | 1.26 | 2.74 | 17 | 18 | 1.06 |
| NAFLD | female | 50 | 5295.628 | 38194.32 | 7.212425 | 26.25 | 4.35 | 1.65 | 1.09 | 2.49 | 15 | 13 | 0.87 |
| NAFLD | male | 50 | 6581.967 | 34905.83 | 5.303252 | 29.07 | 4.42 | 1.23 | 1.29 | 2.47 | 14 | 15 | 1.07 |
| NAFLD | male | 50 | 6840.437 | 36056.8 | 5.271125 | 25.23 | 4.05 | 0.94 | 1.1 | 2.43 | 20 | 28 | 1.4 |
| NAFLD | male | 50 | 7573.77 | 24053.81 | 3.175936 | 27.63 | 4.89 | 1.31 | 1.41 | 2.65 | 35 | 21 | 0.6 |
| NAFLD | female | 50 | 11551.91 | 34517.19 | 2.988007 | 22.9 | 4.59 | 2.74 | 0.99 | 2.14 | 10 | 22 | 2.2 |
| NAFLD | male | 50 | 17366.12 | 24098.65 | 1.387682 | 30.64 | 4.6 | 1.47 | 0.82 | 2.71 | 56 | 34 | 0.61 |
| NAFLD | male | 51 | 8513.661 | 21049.33 | 2.472418 | 25.82 | 5.27 | 1.65 | 0.97 | 3.35 | 24 | 17 | 0.71 |
| NAFLD | female | 51 | 9191.257 | 32798.21 | 3.568414 | 26.47 | 5.16 | 2.72 | 1.68 | 2.37 | 17 | 16 | 0.94 |
| NAFLD | male | 51 | 9286.885 | 42484.3 | 4.574656 | 23.93 | 4.45 | 6.27 | 0.83 | 1.34 | 21 | 20 | 0.95 |
| NAFLD | male | 51 | 9425.137 | 40316.89 | 4.277592 | 26.52 | 4.14 | 2.42 | 0.94 | 2.23 | 30 | 21 | 0.7 |
| NAFLD | male | 51 | 9584.699 | 23814.65 | 2.484653 | 29.86 | 5.1 | 1.42 | 0.96 | 3.01 | 31 | 21 | 0.68 |
| NAFLD | male | 51 | 10000 | 32170.4 | 3.21704 | 24.49 | 5.92 | 1.39 | 1.05 | 3.96 | 26 | 17 | 0.65 |
| NAFLD | male | 51 | 11985.79 | 47656.2 | 3.976058 | 36.36 | 5.81 | 1.57 | 1.15 | 3.69 | 39 | 28 | 0.72 |
| NAFLD | male | 52 | 3426.23 | 35234.68 | 10.2838 | 26.45 | 8.42 | 3.65 | 1.29 | 4.92 | 14 | 15 | 1.07 |
| NAFLD | male | 52 | 6046.995 | 36639.76 | 6.059168 | 25.41 | 4.45 | 0.92 | 1.29 | 2.57 | 22 | 18 | 0.82 |
| NAFLD | male | 52 | 6071.038 | 35488.79 | 5.845589 | 26.79 | 3.92 | 1.14 | 1.01 | 2.3 | 25 | 20 | 0.8 |
| NAFLD | male | 52 | 7180.328 | 19644.25 | 2.735843 | 23.91 | 6.76 | 2.72 | 1.05 | 3.73 | 32 | 22 | 0.69 |
| NAFLD | male | 52 | 10416.94 | 41991.03 | 4.031033 | 28.97 | 6.12 | 6.16 | 1.08 | 2.19 | 43 | 36 | 0.84 |
| NAFLD | male | 52 | 10491.8 | 43994.02 | 4.193181 | 27.99 | 4.91 | 1.83 | 0.99 | 2.79 | 29 | 22 | 0.76 |
| NAFLD | female | 53 | 6708.197 | 31721.97 | 4.728837 | 25.24 | 4.67 | 1.05 | 1.15 | 2.91 | 10 | 14 | 1.4 |
| NAFLD | male | 53 | 7672.131 | 18254.11 | 2.379275 | 22.37 | 4.33 | 1.94 | 0.83 | 2.66 | 13 | 13 | 1 |
| NAFLD | male | 53 | 13726.78 | 33321.38 | 2.427472 | 25.63 | 6.78 | 4.72 | 0.99 | 3.23 | 21 | 19 | 0.9 |
| NAFLD | female | 53 | 14185.79 | 32349.78 | 2.280436 | 21.99 | 6.72 | 2.24 | 1.23 | 3.2 | 19 | 17 | 0.89 |
| NAFLD | female | 53 | 16087.43 | 35518.68 | 2.207853 | 25.16 | 5.92 | 0.61 | 2.34 | 2.88 | 12 | 17 | 1.42 |
| NAFLD | female | 53 | 19081.97 | 44696.56 | 2.342345 | 23.09 | 5.31 | 1.01 | 1.54 | 2.88 | 19 | 21 | 1.11 |
| NAFLD | male | 54 | 2921.311 | 36400.6 | 12.46036 | 25.58 | 4.56 | 1.18 | 1.61 | 2.28 | 21 | 18 | 0.86 |
| NAFLD | female | 54 | 4484.153 | 29136.02 | 6.497553 | 25.36 | 6.17 | 1.48 | 1.33 | 3.78 | 29 | 28 | 0.97 |
| NAFLD | female | 54 | 5650.273 | 42887.89 | 7.59041 | 30.06 | 4.8 | 0.59 | 1.41 | 2.91 | 15 | 16 | 1.07 |
| NAFLD | male | 54 | 8677.596 | 29973.09 | 3.454078 | 25.34 | 3.68 | 0.54 | 1.35 | 1.84 | 30 | 23 | 0.77 |
| NAFLD | female | 54 | 8928.962 | 43814.65 | 4.907026 | 22.43 | 4.33 | 0.96 | 1.33 | 2.31 | 10 | 15 | 1.5 |
| NAFLD | male | 54 | 11661.2 | 24950.67 | 2.139631 | 22.06 | 5.43 | 1.01 | 1.17 | 3.22 | 20 | 17 | 0.85 |
| NAFLD | male | 54 | 11781.42 | 20825.11 | 1.767623 | 24.29 | 6.59 | 1.99 | 1.1 | 3.68 | 12 | 17 | 1.42 |
| NAFLD | male | 54 | 12633.88 | 17581.46 | 1.391612 | 23.22 | 4.93 | 1.43 | 1.01 | 2.8 | 30 | 29 | 0.97 |
| NAFLD | male | 54 | 15978.14 | 22005.98 | 1.377255 | 29.23 | 3.53 | 2.68 | 1.37 | 1.14 | 15 | 17 | 1.13 |
| NAFLD | female | 55 | 677.596 | 42813.15 | 63.18389 | 24.76 | 4.55 | 0.81 | 1.5 | 2.33 | 19 | 22 | 1.16 |
| NAFLD | male | 55 | 3113.661 | 40615.84 | 13.0444 | 30.78 | 5.43 | 1.93 | 1.13 | 3.32 | 41 | 38 | 0.93 |
| NAFLD | male | 55 | 9310.929 | 38956.65 | 4.18397 | 27.48 | 5.19 | 3.37 | 1.01 | 2.79 | 33 | 24 | 0.73 |
| NAFLD | male | 55 | 9540.984 | 21856.5 | 2.290801 | 28.54 | 4.2 | 1.89 | 1.24 | 1.84 | 39 | 24 | 0.62 |
| NAFLD | female | 55 | 10480.87 | 34771.3 | 3.317597 | 24.89 | 5.55 | 2.21 | 1.14 | 3.23 | 20 | 16 | 0.8 |
| NAFLD | male | 55 | 13704.92 | 25115.1 | 1.832561 | 25.68 | 5.44 | 1.17 | 1.05 | 3.12 | 19 | 22 | 1.16 |
| NAFLD | male | 55 | 16743.17 | 43082.21 | 2.573121 | 20.66 | 6.55 | 6.17 | 1 | 2.23 | 48 | 21 | 0.44 |
| NAFLD | male | 56 | 5644.262 | 37641.26 | 6.668943 | 28.83 | 4.55 | 2.82 | 1.27 | 2.17 | 15 | 20 | 1.33 |
| NAFLD | male | 56 | 6569.945 | 35324.36 | 5.37666 | 24.6 | 5.61 | 1.04 | 1.14 | 3.56 | 53 | 37 | 0.7 |
| NAFLD | male | 56 | 7032.787 | 45518.68 | 6.472353 | 29.94 | 5.2 | 1.27 | 1.03 | 3.22 | 17 | 14 | 0.82 |
| NAFLD | male | 56 | 8437.158 | 22768.31 | 2.698576 | 29.21 | 4.71 | 2.6 | 1.18 | 2.41 | 34 | 26 | 0.76 |
| NAFLD | male | 56 | 8896.175 | 36968.61 | 4.155562 | 35.36 | 3.91 | 0.95 | 1.18 | 2.03 | 33 | 29 | 0.88 |
| NAFLD | male | 56 | 9748.634 | 43994.02 | 4.512839 | 27.31 | 4.74 | 2.78 | 1.35 | 2.21 | 18 | 21 | 1.17 |
| NAFLD | male | 56 | 11617.49 | 21841.55 | 1.880058 | 26.43 | 4.36 | 3.61 | 1.05 | 1.79 | 39 | 25 | 0.64 |
| NAFLD | male | 56 | 15879.78 | 21976.08 | 1.383903 | 28.85 | 4.98 | 1.66 | 1.69 | 2.36 | 24 | 24 | 1 |
| NAFLD | male | 57 | 6028.962 | 39733.93 | 6.590509 | 27.81 | 4 | 1.78 | 0.78 | 2.4 | 39 | 23 | 0.59 |
| NAFLD | male | 57 | 7417.486 | 41617.34 | 5.610707 | 26.52 | 4.25 | 2.96 | 0.97 | 1.7 | 29 | 24 | 0.83 |
| NAFLD | female | 57 | 8644.809 | 38911.81 | 4.501176 | 21.52 | 5.85 | 2.43 | 1.05 | 3.2 | 20 | 19 | 0.95 |
| NAFLD | male | 58 | 5868.852 | 22170.4 | 3.777638 | 26.33 | 3.94 | 1.54 | 0.94 | 2.01 | 19 | 21 | 1.11 |
| NAFLD | female | 58 | 8765.027 | 21004.48 | 2.396396 | 26.51 | 6.62 | 2.72 | 1.09 | 3.74 | 28 | 20 | 0.71 |
| NAFLD | male | 58 | 9704.918 | 36819.13 | 3.793863 | 23.83 | 6.3 | 1.93 | 1.22 | 3.09 | 27 | 23 | 0.85 |
| NAFLD | female | 58 | 11366.12 | 30855.01 | 2.714648 | 24.11 | 7.21 | 2.07 | 1.25 | 4.52 | 13 | 19 | 1.46 |
| NAFLD | female | 58 | 12426.23 | 21662.18 | 1.743262 | 26.41 | 4.6 | 1.54 | 1.01 | 2.68 | 28 | 20 | 0.71 |
| NAFLD | female | 59 | 6353.552 | 45294.47 | 7.129 | 25.34 | 3.44 | 0.99 | 0.84 | 1.98 | 11 | 15 | 1.36 |
| NAFLD | male | 59 | 6743.169 | 32349.78 | 4.797415 | 23.91 | 4.34 | 2.15 | 0.76 | 2.41 | 25 | 16 | 0.64 |
| NAFLD | male | 59 | 9449.18 | 42409.57 | 4.488175 | 25.57 | 5.98 | 3.58 | 1.43 | 3.17 | 19 | 22 | 1.16 |
| NAFLD | male | 59 | 10043.72 | 29718.98 | 2.958961 | 24.92 | 5.02 | 1.3 | 1.08 | 3.01 | 39 | 29 | 0.74 |
| NAFLD | female | 59 | 10109.29 | 25488.79 | 2.521323 | 25.38 | 6.06 | 1.95 | 1 | 3.66 | 18 | 13 | 0.72 |
| NAFLD | male | 59 | 12218.58 | 23904.33 | 1.956392 | 24.62 | 5.62 | 3.22 | 1.07 | 2.74 | 122 | 85 | 0.7 |
| NAFLD | male | 60 | 7748.634 | 29584.45 | 3.818021 | 30.43 | 5.18 | 2.87 | 0.85 | 2.79 | 24 | 21 | 0.88 |
| NAFLD | male | 60 | 11945.36 | 21527.65 | 1.802177 | 27.29 | 5.73 | 1.42 | 1.25 | 3.35 | 20 | 18 | 0.9 |
| NAFLD | female | 61 | 10830.6 | 21213.75 | 1.958686 | 27.7 | 4.43 | 4.94 | 0.88 | 1.64 | 11 | 14 | 1.27 |
| NAFLD | female | 61 | 12251.37 | 31124.07 | 2.540456 | 28.03 | 5.63 | 2.52 | 1.13 | 3.05 | 37 | 21 | 0.57 |
| NAFLD | male | 62 | 8568.306 | 22035.87 | 2.571788 | 22.75 | 4.91 | 1.11 | 1.75 | 2.3 | 14 | 12 | 0.86 |
| NAFLD | male | 62 | 11147.54 | 40780.27 | 3.65823 | 29.93 | 4.91 | 1.26 | 0.88 | 3.28 | 50 | 27 | 0.54 |
| NAFLD | male | 64 | 5956.831 | 36550.07 | 6.135825 | 22.57 | 5.11 | 0.62 | 2.31 | 2.1 | 6 | 18 | 3 |
| NAFLD | male | 64 | 6473.77 | 41019.43 | 6.336251 | 27.15 | 4.01 | 1.35 | 1.17 | 2.18 | 100 | 63 | 0.63 |
| NAFLD | female | 64 | 6928.962 | 38508.22 | 5.557574 | 27.62 | 7.57 | 2.09 | 1.56 | 4.61 | 18 | 15 | 0.83 |
| NAFLD | male | 64 | 7736.066 | 33695.07 | 4.355582 | 24.64 | 5.98 | 1.76 | 1.29 | 3.58 | 14 | 15 | 1.07 |
| NAFLD | male | 64 | 13890.71 | 18373.69 | 1.322732 | 26.87 | 5.17 | 3.56 | 0.95 | 2.04 | 30 | 30 | 1 |
| NAFLD | male | 65 | 5974.863 | 38000 | 6.359978 | 24.03 | 4.93 | 1.87 | 1.6 | 2.37 | 27 | 29 | 1.07 |
| NAFLD | female | 65 | 10316.94 | 36819.13 | 3.568803 | 25.77 | 3.45 | 2.09 | 1.04 | 1.42 | 18 | 21 | 1.17 |
